# Transcatheter Aortic Valve Replacement for Severe Aortic Regurgitation in a Young Adult With Takayasu Arteritis: A Case Report

**DOI:** 10.7759/cureus.108177

**Published:** 2026-05-03

**Authors:** Janhvi Shah, Donica Baker

**Affiliations:** 1 Internal Medicine, St. Luke's Hospital, Chesterfield, USA; 2 Rheumatology, St. Luke's Hospital, Chesterfield, USA

**Keywords:** aortic regurgitation, multidisciplinary care, takayasu arteritis, transcatheter aortic valve replacement

## Abstract

Takayasu arteritis (TAK) is a rare large-vessel vasculitis that can cause severe aortic regurgitation (AR) and heart failure in young adults. While surgical aortic valve replacement remains the standard treatment, transcatheter aortic valve replacement (TAVR) has emerged as a viable alternative in high-risk patients. We present the case of a 23-year-old woman with juvenile idiopathic arthritis (JIA) and TAK who developed refractory heart failure secondary to severe AR. Initial echocardiography revealed a left ventricular ejection fraction (LVEF) of 40%. Given active vasculitis, prohibitive surgical risk, and acute decompensation despite medical therapy, emergent TAVR was performed. The patient demonstrated remarkable clinical improvement with LVEF normalization to 56% at one-year follow-up. This case illustrates that TAVR can be successfully performed for severe AR in young adults with TAK when surgical risk is prohibitive, emphasizing the importance of aggressive perioperative inflammation control, multidisciplinary care, and individualized treatment strategies in vasculitis-associated valvular disease.

## Introduction

Takayasu arteritis (TAK) is a rare, chronic, granulomatous large-vessel vasculitis predominantly affecting the aorta and its major branches, with a marked predilection for young women and typically with onset in childhood or early adulthood [1]. The clinical spectrum of TAK is heterogeneous, ranging from nonspecific constitutional symptoms to catastrophic vascular complications, including stenosis, aneurysm formation, and the destruction of the aortic wall leading to aortic regurgitation (AR) and heart failure [1,2]. Childhood-onset TAK (age of onset <16 years) is associated with systemic inflammation and widespread vascular involvement, but a lower incidence of cardiac complications, including moderate-to-severe aortic insufficiency and left ventricular dysfunction [3].

The overlap of TAK with juvenile idiopathic arthritis (JIA) is rare but clinically significant given the increased long-term cardiovascular risk, including accelerated atherosclerosis and valvular disease, associated with both conditions [4,5]. AR in TAK results from chronic inflammatory destruction of the aortic root and valve, leading to progressive left ventricular dilation and dysfunction and ultimately heart failure [2]. The American College of Cardiology/American Heart Association (ACC/AHA) guidelines define symptomatic severe AR with left ventricular ejection fraction (LVEF) below 55% as an indication for urgent intervention, not just specifically for the aortitis population, given the risk of irreversible myocardial damage [6,7].

While surgical aortic valve replacement (SAVR) remains the standard of care [8], it carries higher rates of prosthetic valve detachment and paravalvular leak due to ongoing aortic inflammation [2,8]. Transcatheter aortic valve replacement (TAVR) has emerged as a viable alternative in select high-risk patients [9-11]. However, data on TAK remain limited to a handful of case reports [10-12].

We report the case of a 23-year-old woman with TAK and JIA, complicated by recurrent pulmonary embolism and severe AR, who presented with acute-on-chronic heart failure. Her management required emergent TAVR, resulting in marked symptomatic and echocardiographic improvement at 12 months. This case adds to the limited literature on TAVR in TAK and underscores the importance of individualized, multidisciplinary management for patients with complex vasculitis.

## Case presentation

A 23-year-old woman with a history of JIA (diagnosed at age 10) and TAK (diagnosed at age 17) presented with progressive exertional dyspnea over several months and three days of hemoptysis. She had a history of recurrent pulmonary embolism (2017 and 2021) and was maintained on apixaban. Prior immunosuppressive regimens had included rituximab, prednisone, mycophenolate mofetil, and azathioprine; she had discontinued all immunosuppression two years prior due to side effects.

On presentation, she was in respiratory distress with an oxygen saturation of 80% on room air. Vital signs revealed a wide pulse pressure (142/58 mmHg), tachycardia (128 beats per minute), and decreased right-sided breath sounds. Laboratory studies demonstrated elevated inflammatory markers (C-reactive protein 8.5 mg/dL; erythrocyte sedimentation rate 114 mm/hr) and a markedly elevated N-terminal pro-B-type natriuretic peptide (NT-proBNP) level of 19,000 pg/mL.

**Figure 1:**
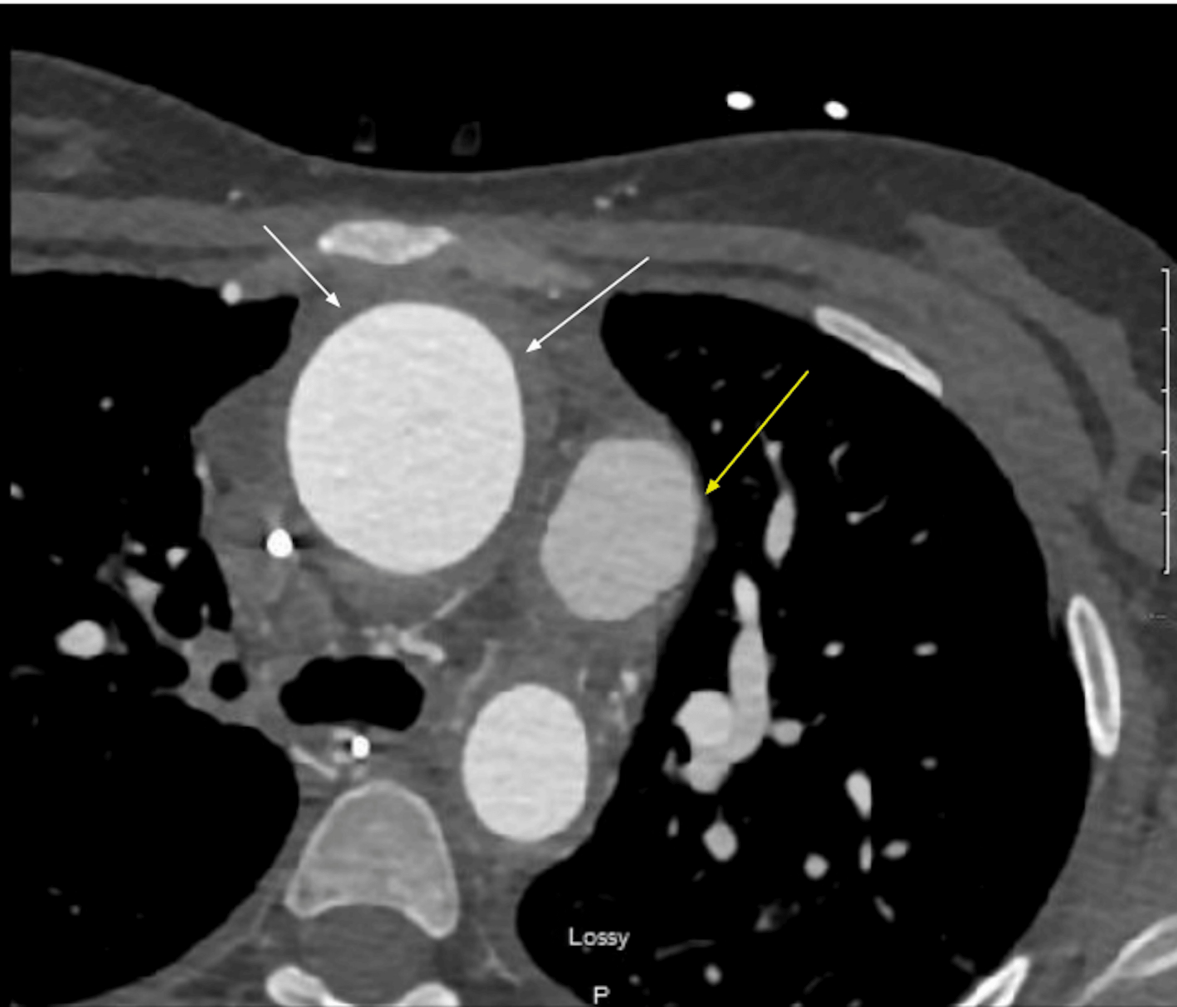
CT angiogram of the chest showing aortic wall thickening The white arrows show the ascending aorta with thickened walls, and the yellow arrow points towards the main pulmonary artery. CT: computed tomography

Computed tomography (CT) angiography (Figure 1) demonstrated circumferential aortic wall thickening with enhancement consistent with active aortitis, aortic root dilation, bilateral carotid occlusion, and right lower lobe pneumonia. These findings confirmed the TAK finding in the patient by fulfilling the required Kerr criteria for disease activity assessment. Transthoracic echocardiography revealed severe aortic insufficiency (proximal isovelocity surface area regurgitant volume 19 mL/beat, vena contracta 0.7 cm), LVEF of 39%, left ventricular dilation (end-diastolic dimension 5.4 cm), and moderate left ventricular hypertrophy. Transesophageal echocardiography confirmed these findings. Cardiac catheterization demonstrated moderate pulmonary hypertension (right ventricular systolic pressure 51 mmHg), severe grade 3 to 4+ AR, and non-obstructive coronary artery disease.

The multidisciplinary heart team deemed surgery as high risk due to active vasculitis based on their clinical judgement. The patient was admitted to the intensive care unit and treated with intravenous diuretics, antibiotics, and methylprednisolone 125 mg daily for three days and then transitioned to oral prednisone 60 mg daily. Tocilizumab 8 mg/kg monthly was initiated. Cardiothoracic surgery deferred valve replacement until disease control was achieved, given the operative risk. On day 10, after clinical stabilization, she was discharged on prednisone 40 mg daily with a planned taper.

**Figure 2:**
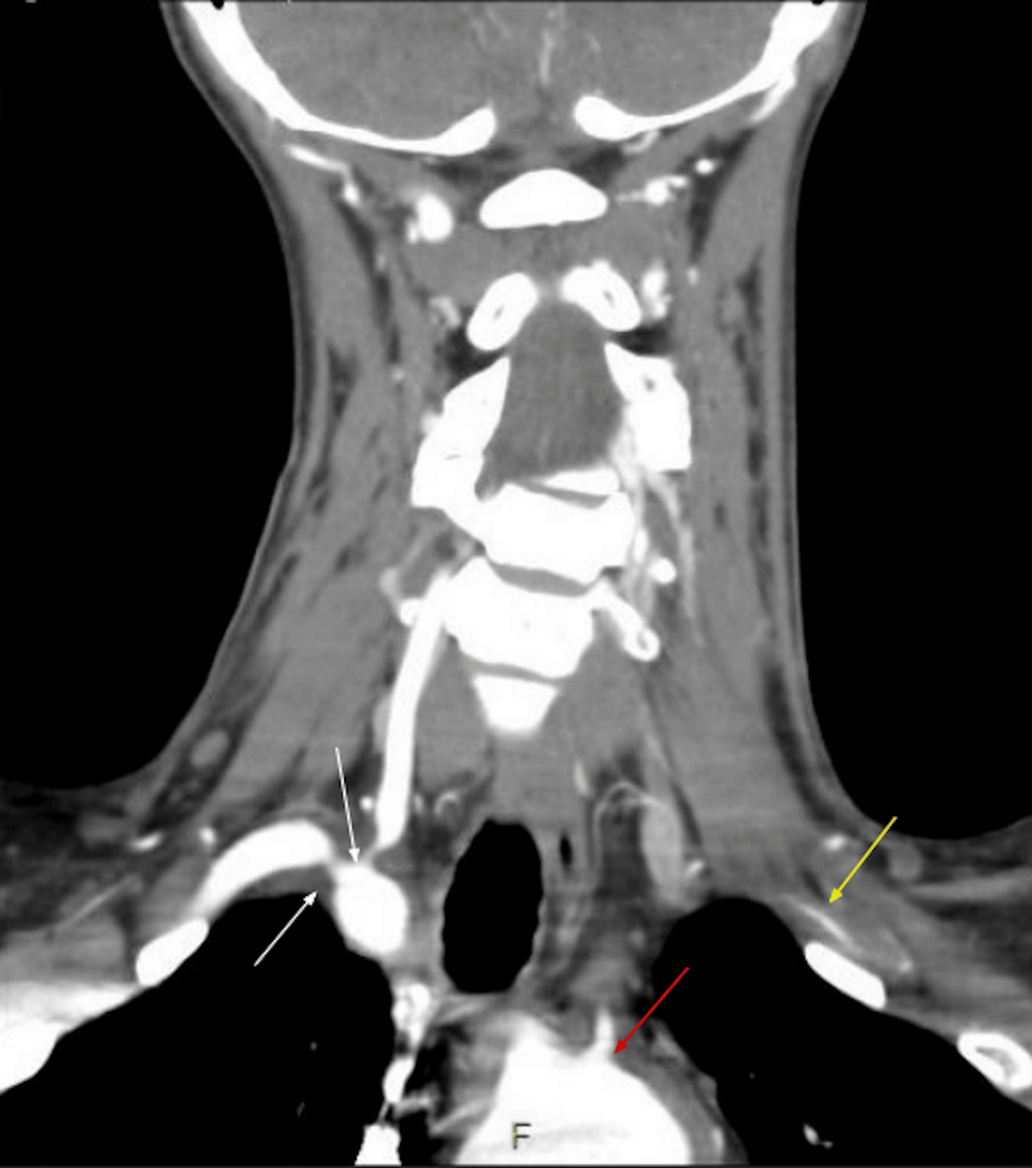
CT angiogram TAVR protocol showing multiple arterial narrowing The white arrows point to the right subclavian artery and original right vertebral artery on the right. The yellow arrow shows the left subclavian artery. The red arrow points towards the aortic arch. CT: computed tomography; TAVR: transcatheter aortic valve replacement

She was evaluated at the outpatient clinic one week later and found to be clinically stable. The following day, however, she returned to the emergency department with severe dyspnea and hypoxia after attending a pool party. At the time of her presentation, she was taking only apixaban. NT-proBNP had risen to 22,500 pg/mL, and the chest radiograph was consistent with acute decompensated heart failure. Within hours, she developed acute-on-chronic respiratory failure requiring emergent endotracheal intubation. Hemoptysis was noted during intubation, and anticoagulation was held indefinitely. Despite vasopressor support and aggressive diuresis, NT-proBNP rose to >25,000 pg/mL, and LVEF declined to 35%. A multidisciplinary consensus recommended emergent TAVR. CT angiography TAVR protocol was performed (Figure 2), which showed narrowing of multiple arteries in the head and neck region.

Under general anesthesia with transesophageal echocardiographic guidance, transfemoral TAVR was performed via the right femoral artery. A 34-mm Medtronic Evolut self-expanding valve was deployed under temporary pacing with combined fluoroscopic and echocardiographic guidance. Post-deployment assessment revealed a well-seated valve with no perivalvular leak and hemodynamic improvement. The procedure was completed without intraoperative complications or transfusions. The self-expanding Evolut design was selected because of the following: (1) its self-expanding design accommodates the dilated aortic roots common in TAK; (2) it provides radial force for anchoring without a calcified substrate; (3) supra-annular positioning minimizes contact with inflamed tissue; and (4) it allows repositioning in challenging anatomy [9,13].

The patient was extubated on postoperative day 1. She developed a right groin hematoma that resolved conservatively; arterial duplex ultrasonography excluded pseudoaneurysm or arteriovenous fistula. In agreement with the European Alliance of Associations for Rheumatology (EULAR) 2018 algorithm-recommended phased approach, immunosuppression was restarted as prednisone 20 mg daily with a planned taper of 5 mg weekly, tocilizumab 480 mg monthly, and mycophenolate mofetil 500 mg twice daily. Intensive medication adherence support was initiated, including pharmacy counseling, visual medication schedules, and weekly follow-up. She was discharged on postoperative day 6.

At the one-week follow-up, she had complete symptom resolution (New York Heart Association class I), and LVEF had improved to 47%. At three months, inflammatory markers had normalized (C-reactive protein 0.4 mg/dL, erythrocyte sedimentation rate 12 mm/hr), and LVEF had improved to 52%. Prednisone was successfully tapered off, and she was transitioned to mycophenolate mofetil 1,000 mg twice daily with continued tocilizumab. At the 12-month follow-up, LVEF had improved to 56% (progressive improvement: 40% pre-TAVR to 47% at one week, 52% at three months, and 56% at 12 months), left ventricular end-diastolic dimension had regressed from 5.4 cm to 4.3 cm, the prosthetic valve was stable with only trivial paravalvular leak and no thrombosis, inflammatory markers remained suppressed, and the patient was working full time in New York Heart Association class I status. This was attributed to the combined management including both valve correction and immunosuppressive therapy, which highlights the importance of multifactorial contribution.

## Discussion

This case demonstrates successful emergent TAVR for severe AR in the youngest reported patient with TAK (age 23) and with the longest published follow-up of 12 months, showing sustained hemodynamic and clinical benefit [10-12].

Only a limited number of prior TAVR cases for TAK-associated AR have been reported in the literature, involving patients aged 40-70 years [10,12]. All demonstrated technical success, supporting TAVR feasibility in this population (Table 1). Our case extends this literature by reporting the youngest patient treated and the longest documented follow-up, showing progressive left ventricular reverse remodeling over 12 months.

**Table 1:** List of cases previously published on TAVR for Takayasu arteritis-associated aortic regurgitation LVEF: left ventricular ejection fraction; NR: not reported; TAVR: transcatheter aortic valve replacement

Study	Year of publication	Mean age (yr)	Mean follow-up	No. of cases included	LVEF pre (%)	LVEF post (%)
Shah and Baker (current)	2026	23	1 year	1	40	56
Tadokoro et al. [12]	2025	67.3	5.7 years	3	NR	NR
Chen et al. [10]	2022	61.2	1 year	5	43.6 ± 8.0	51.4 ± 10.2

The pathogenesis of severe AR in TAK involves chronic granulomatous inflammation of the aortic root and valve, leading to progressive left ventricular dilation and dysfunction [1,2]. SAVR remains guideline-recommended for severe symptomatic AR with left ventricular dysfunction [6,7], but carries heightened risk in TAK due to annular fragility, prosthetic detachment, and paravalvular leak from inflamed tissue [2,8]. TAVR for native AR is technically challenging owing to the absence of a calcified landing zone to anchor the prosthesis; however, contemporary self-expanding devices that rely on radial force rather than calcification for anchoring appear better suited to the dilated, non-calcified aortic roots characteristic of TAK [9,13]. The ACC/AHA guidelines acknowledge TAVR for isolated AR as an option in carefully selected patients when surgical risk is prohibitive [6].

A critical learning point from this case is the role of active vasculitis as a driver of TAVR outcomes. Our patient's CT angiography demonstrated circumferential aortic wall thickening with enhancement, and elevated inflammatory markers confirmed active disease at initial presentation. Aggressive perioperative immunosuppression with high-dose corticosteroids combined with tocilizumab, an interleukin-6 receptor antagonist with established efficacy in refractory TAK [14], was central to achieving the favorable result. Normalization of inflammatory markers by three months correlated with sustained clinical improvement and supports the strategy of combining biologic disease control with transcatheter valve intervention.

This case also highlights medication non-adherence as a potentially life-threatening precipitant in vasculitis. Premature discontinuation of immunosuppression might have caused a rebound inflammation and precipitated the acute decompensation leading to emergent TAVR. Intensive adherence interventions during hospitalization, including proactive side-effect management, pharmacy counseling, visual schedules, and weekly multidisciplinary follow-up, were followed by sustained adherence at 12 months and successful steroid taper. The importance of multidisciplinary surveillance of both disease activity and valve function has been emphasized in ACC/AHA and the American College of Rheumatology (ACR)/Vasculitis Foundation guidelines [6,14,15].

Limitations of this report include its single-center, single-patient design and the 12-month follow-up horizon, which is insufficient to characterize long-term prosthetic durability in a 23-year-old patient. Questions regarding valve lifespan, the feasibility of valve-in-valve procedures, and optimal immunosuppression protocols in this population require prospective multicenter registry data.

## Conclusions

TAVR can be performed successfully for severe AR in young adults with TAK when the surgical risk is prohibitive. At the 12-month follow-up, our patient achieved LVEF improvement from 40% to 56%, significant left ventricular reverse remodeling, and New York Heart Association class I functional status. Key success factors were appropriate patient selection, recognizing prohibitive surgical risk, choice of a self-expanding valve suited to the dilated non-calcified aortic root, aggressive perioperative inflammation control with biologic therapy, proactive medication adherence support to prevent immunosuppression-related relapse, and multidisciplinary team collaboration. Continuous surveillance for aortic valve involvement is warranted in all TAK patients. As transcatheter technologies continue to evolve, prospective registries are needed to refine patient selection criteria, define optimal immunosuppression protocols, and establish long-term prosthetic durability in vasculitis-associated valvular disease.
